# Improving Bioaccessibility and Bioavailability of Isoflavone Aglycones from Chickpeas by Germination and Forming β-Cyclodextrin Inclusion Complexes

**DOI:** 10.3390/pharmaceutics15122684

**Published:** 2023-11-27

**Authors:** Yuanfan He, Jiani Xiang, Jie Chen, Sheng Fang, Zili Guo, Xianrui Liang

**Affiliations:** 1 College of Pharmaceutical Sciences, Zhejiang University of Technology, Hangzhou 310014, China; 15988802063@163.com (Y.H.); xiangjiani1226@163.com (J.X.); 2 School of Food Science and Biotechnology, Zhejiang Gongshang University, Hangzhou 310018, China; chenjie@zjgsu.edu.cn (J.C.); fszjgsu@163.com (S.F.); 3 Key Laboratory of Pollution Exposure and Health Intervention of Zhejiang Province, Interdisciplinary Research Academy (IRA), Zhejiang Shuren University, Hangzhou 310015, China

**Keywords:** chickpea isoflavones, cyclodextrin inclusion, pharmacokinetics

## Abstract

Chickpea isoflavones have diverse pharmacological activities but with low water solubility and bioavailability. In this work, the isoflavone content in chickpeas was first increased by germination, and then the bioaccessibility and bioavailability of isoflavones in chickpea sprout extracts (CSE) were enhanced using β-cyclodextrin (β-CD) inclusion techniques. Firstly, the total content of isoflavones was increased by 182 times through sprouting, and isoflavones were presented mostly in the germ and radicle. Then, the chickpea sprout extract/β-cyclodextrin (CSE/β-CD) inclusion complex was prepared and characterized. The in vitro test showed that the cumulative release of two isoflavones, formononetin (FMN) and biochanin A (BCA), in the CSE/β-CD was significantly increased in a simulated digestive fluid. The in vivo rat pharmacokinetics demonstrated that the inclusion of FMN and BCA by β-CD effectively increased their bioavailability in rat plasma and tissues, especially in the liver. The study provides a feasible strategy for improving the bioavailability of isoflavones from chickpeas and is also beneficial to the utilization of other legume resources.

## 1. Introduction

Chickpea (*Cicer arietinum* Linn.) originated in western Asia and the Near East and has a long history of cultivation. It ranks third in the world as the most important food legume [1]. It contains many healthful constituents including carbohydrates, protein, fiber, fat, and a variety of active ingredients [2]. Chickpea is also widely used in traditional medicine to prevent and treat various diseases like cardiovascular disease, hyperlipidemia, diabetes, stomach disease, etc., due to the abundant content of isoflavone constituents [3].

Isoflavones in beans are a class of important secondary metabolites with a wide range of pharmacological activities, such as an estrogen-like effect, hypoglycemic, hypolipidemic, anti-inflammatory, anti-virus, anti-tumor, the prevention and treatment of osteoporosis caused by estrogen deficiency, the treatment of cardiovascular diseases and neurodegenerative diseases, etc [4]. However, the content of isoflavones in untreated beans is relatively low but can be increased by sprouting treatment [5,6]. In germinated chickpeas, the content of isoflavones is increased by more than 100 times as compared to untreated chickpeas, which are a good source of isoflavones [7].

On the other hand, isoflavones have a large planar conjugated system with a short distance between molecules and a tight structure, which makes it difficult for the solvent to disperse these molecules, resulting in poor water solubility and low bioavailability [8,9]. Many methods are devoted to improving their water solubility and bioavailability including cyclodextrin (CD) inclusion [10]. CDs have a unique structural characteristic of “external hydrophilicity and internal hydrophobicity”, which enables the selective inclusion of guest small molecules to improve their water solubility [11]. Studies showed that CDs had a good effect on improving the solubility of flavonoid compounds [12,13]. For example, Yang et al. improved the water solubility of taxifolin, quercetin, and morin hydrate by preparing propylenediamine-modified β-CD inclusion complexes [12]. In our previous work, we prepared a formononetin/methyl-β-CD inclusion complex to improve the solubility of formononetin (FMN) by approximately 50 times [13]. Chickpea is rich in isoflavones including FMN and biochanin A (BCA), but the poor solubility limits its pharmacological activity in vivo. At present, the number of studies on the solubilization, bioaccessibility, and bioavailability improvement of chickpea isoflavones is lower, which limits the further development of chickpea isoflavones functional products.

In this study, the content changes in isoflavones in chickpeas during germination and isoflavones distribution in different sprout parts were investigated and the chickpea sprout extract (CSE) was prepared. Then the β-CD/CSE inclusion complex was prepared and characterized. The in vitro simulated gastrointestinal release and the in vivo pharmacokinetics and tissue distribution of the β-CD/CSE inclusion complex were studied. It is known that most of the absorbed and active forms of chickpea isoflavones in the body are in the aglycone form [2,3]. Therefore, two isoflavone aglycones (BCA and FMN) were chosen as target compounds for inclusion complex characterization and pharmacokinetic studies. This investigation provides significant research data for the comprehensive utilization of chickpeas and isoflavones. It also provides method references both for the in vitro bioaccessibility and in vivo bioavailability inclusion complex of plant extracts.

## 2. Materials and Methods

### 2.1. Materials and Reagents

Chickpeas were obtained from Mulei County, Xinjiang, China. FMN (98%), BCA (98%), chloramphenicol (CHL, 98%), and USP-grade pepsin and trypsin were from Aladdin (Shanghai, China). The Acetonitrile and formic acid of HPLC grade were purchased from Merck (Darmstadt, Germany) and Macklin (Shanghai, China), respectively. The β-cyclodextrin (β-CD) was purchased from Sigma Aldrich (Shanghai, China). Other analytical-grade reagents were purchased from commercial sources.

### 2.2. Germination of Chickpeas and Preparation of Chickpea Sprout Extract (CSE)

Chickpeas were soaked in water for 12 h, then put into the germination machine for cultivation, and incubated at 25 °C for 10 days without light. Every day, 40 germinated seeds were collected from day 0 to day 10, ground into powder after drying (60 °C, 10 h), and soaked in petroleum ether for 24 h. After removing the petroleum ether, the powder was extracted twice by a 60% ethanol aqueous solution at the ratio of 1:20 (g/mL) and sonicated for 30 min at 40 °C. After centrifuging at 10,000 rpm for 10 min, the supernatant was evaporated and freeze-dried to obtain CSE.

### 2.3. Identification and Quantification of Isoflavones

Thermo Scientific UltiMate 3000 UHPLC coupled to the LCQ FLEET IT-MS system (Waltham, MA, USA) was applied to the component analysis of CSE. The detection wavelength was set at 254 nm. A Welch Ultimate XB-C18 (250 mm × 4.6 mm, 5 μm) column was used at 30 °C and the mobile phase was 0.05% formic acid solution (A) and acetonitrile (B). The gradient elution was 0–30 min, 82–32% A; 30–35 min, 32–20% A; 35–36 min, 20–82% A; 36–40 min, 82% A. A 1.0 mL/min flow rate was used and 10 μL of the sample was injected. The capillary temperature and voltage of mass spectrometry were 300 °C and 11.5 V, respectively. The sheath and auxiliary gas were Nitrogen with a spray voltage of 4 kV. The ionization mode was positive with a mass scan range of 50 to 1200 (*m*/*z*). An Agilent 1260 Infinity II HPLC with a DAD detector (Palo Alto, CA, USA) was used for the quantification of isoflavones in CSE. The HPLC conditions were identical to the above UHPLC analysis conditions.

For the quantification of isoflavones, the FMN and BCA standards were used to prepare the series of methanol solutions with concentrations of 0.25, 2.5, 5, 20, 40, 60, 80, and 100 μg/mL, respectively. The solutions were determined by HPLC to establish two standard curves for the content determination of these two isoflavones in chickpea sprouts.

### 2.4. Determination of Total Flavonoids in CSE

The AlCl_3_ chromogenic method was used to determine the total flavonoids in CSE by using reference standard BCA to establish the standard curve [14]. BCA was weighed to make a concentration of 11.26 μg/mL of methanol solution. Then 1.00, 2.00, 4.00, 6.00, and 8.00 mL of this solution were respectively placed into the 10 mL volumetric flask, adding 1 mL of the 0.1 mol/L AlCl_3_ solution and diluting to the scale using methanol to obtain a series of solutions (1.13-9.01 μg/mL). After 30 min, the absorbance determination was carried out on a UV spectrophotometer (Shimadz UV-2250, Kyoto, Japan) at 273 nm. The content of total flavonoids in the CSE was presented as BCA equivalents (mg BCA/g CSE).

### 2.5. Inclusion of Chickpea Sprout Isoflavones

A saturated aqueous solution method was used to prepare the inclusion complexes [15]. β-CD (456 mg) was dispersed into 20 mL of water and heated with a water bath. CSE was accurately weighed according to the mass ratio of β-CD/CSE at 7:1 (g/g) to 3:1 (g/g) and dissolved in a small amount of ethanol to obtain CSE ethanol suspension, which was slowly dripped into the β-CD aqueous solution under 30 to 70 °C and at a stirring speed of 200 rpm to 1000 rpm. The mixed solution was stirred for 0.5 h to 4 h. After cooling to room temperature, the mixture was placed at 4 °C for 24 h, and then filtered quickly. The filter residue was moistened with a small amount of ethanol and dried to obtain the β-CD/CSE inclusion complex. The amounts of BCA and FMN in the initial CSE (*M_c_*) and filtrate (*M_f_*) were determined under the same conditions as above using an Agilent 1260 Infinity II HPLC. The inclusion ratio of FMN and BCA was used as an evaluation index to optimize the preparation methods. The calculation formula of the inclusion ratio (*R*%) was as follows:

(1) $$R\%=\left(1-M_{f}\right)/M_{c}\times 100\%$$

where *M_f_* is the mass of the remaining isoflavones in the filtrate and *M_c_* is the mass of the isoflavones in the CSE initially added.

### 2.6. Characterization of β-CD/CSE Inclusion Complex

#### 2.6.1. UV-Vis Analysis

The absorbances of β-CD, CSE, the physical mixture of CSE/β-CD, and the CSE/β-CD inclusion complex were measured on a Shimadzu UV-2250 UV-vis spectrophotometer (Kyoto, Japan) ranging from 200 to 400 nm. All the samples were dissolved in 10 mL of water to obtain four sample solutions with a concentration of 1.00 mg/mL, respectively.

#### 2.6.2. FT-IR Analysis

A Thermo Scientific Nicolet iS50 FT-IR spectrometer (Waltham, America) was applied to collect the Fourier-transform infrared (FT-IR) spectra of β-CD, CSE, the physical mixture of CSE/β-CD, and the CSE/β-CD inclusion complex using KBr (sample: KBr (*w*/*w*) = 1:30) to prepare sample disks. Absorption ranging from 4000–400 cm^−1^ was recorded.

#### 2.6.3. SEM Analysis

The surface morphology of β-CD, CSE, the physical mixture of CSE/β-CD, and the CSE/β-CD inclusion complex was determined on a Hitachi S-4700 scanning electron microscope (SEM) (Tokyo, Japan). The accelerating voltage was 15.0 kV with a magnification of 2000. The samples were evenly dispersed with the conductive tape coating with a platinum layer for 200 s.

### 2.7. In Vitro Sustained Release in Simulated Digestion Model

According to the determination method of dissolution and release in Chinese Pharmacopoeia 2020, the in vitro sustained release of the isoflavones in the CSE/β-CD inclusion complex and CSE was determined on a SOTAX AT. 7× dissolution tester (Stetten, Switzerland) with a stirring rate of 100 rpm at 37 °C [16] using FMN and BCA as the evaluation index. Simulated gastric fluid (SGF) and simulated intestinal fluid (SIF) were used as release media. The preparation method of SGF was to mix 16.4 mL of the HCl solution (9.5–10.5% by volume) and 10 g of pepsin into 800 mL water, and finally adjust the volume to 1000 mL of water. The preparation of SIF was to add 6.8 g of potassium dihydrogen phosphate to 500 mL of water (use 0.1 M NaOH to adjust the pH to 6.8), then add 10 g of trypsin, and finally adjust the volume to 1000 mL of water. The CSE/β-CD inclusion complex (400.00 mg, containing 2.08 mg FMN and 7.16 mg BCA), CSE (94.12 mg, containing 2.08 mg FMN), and CSE (205.16 mg, containing 7.16 mg BCA) were weighed and added to 900 mL of release media, respectively. At 0.17, 0.33, 0.50, 0.75, 1, 1.5, 2, 3, 4, 5, 6, 8, 10, 12, and 24 h, samples (2 mL) were respectively extracted and replaced with an equal volume of the same release medium. The sample was added to 3 times the volume of acetonitrile to remove the protein, then centrifuged at 10000 rpm for 10 min, and the obtained supernatant was analyzed by HPLC. The calculation formula for the cumulative percentage release (*C%*) was as below:

(2) $$C\%=M_{t}/M_{0}\times 100\%$$

where *M_t_* is the total mass of isoflavones released cumulatively at each time point and *M*_0_ is the total mass of isoflavones initially added.

### 2.8. Animal Experiments

The specific pathogen-free (SPF) male Sprague–Dawley (SD) rats (average body weight, 220 ± 10 g) aged eight weeks old were purchased from Shanghai Slake Laboratory Animal Co., Ltd. (Shanghai, China). After being raised in an environment-controlled feeding room for 1 week, the SD rats were divided into three groups (groups A, B, and C), with six in each group. They were fasted for 12 h and supplied with unlimited water before the experiments. In group A, 200 mg of the CSE/β-CD inclusion complex (FMN: 1.04 mg, BCA: 3.58 mg) was weighed and prepared into a 3 mL suspension by pure water and then given to each rat by gavage. In group B, a certain amount of CSE with FMN of the same quality as that in the inclusion complex was weighed and prepared into a 3 mL suspension by pure water and then given to each rat by gavage. In group C, a certain amount of CSE, ensuring that the content of BCA was the same as that in the inclusion complex, was weighed and prepared into s 3 mL suspension by pure water and then given to each rat by gavage. The dosage of FMN and BCA in rats was controlled at approximately 5 mg/kg and 18 mg/kg, respectively. Approximately 300 μL rat retro-orbital blood samples were obtained and transferred to heparinized tubes before dosing at 0.083, 0.25, 0.50, 0.75, 1, 2, 4, 6, 8, 12, and 24 h following intragastric administration. After centrifuging at 3500 rpm for 10 min, the upper plasma was put in clean tubes and placed at −20 °C before use. The rats were killed using the carbon dioxide box after taking blood at the last time point. A batch of new SD rats was raised with the same grouping and administration method for the study of tissue composition. At 0.25 h after intragastric administration, the rats were killed, and the organs including hearts, livers, spleens, lungs, kidneys, and brains were quickly separated, put into centrifuge tubes, and temporarily stored at 4 °C.

The animal care procedures and protocols were conducted following the Guide for the Care and Use of Laboratory Animals in the Zhejiang University of Technology (MGS20220914001) while conforming to the National Institutes of Health Guide for Care and Use of Laboratory Animals (Publication No. 85-23, revised 1996).

### 2.9. Pharmacokinetic Study of CSE and β-CD/CSE Inclusion Complex

#### 2.9.1. Chromatographic and Mass Spectrometry Conditions

To comparatively evaluate the pharmacokinetics of the isoflavones in CSE and the CSE/β-CD inclusion complex, the content of FMN and BCA in rat plasma was determined on a UPLC-MS/MS system equipped with a Waters ACQUITY UPLC and a Xevo TQ-S micro ESI-MS/MS system (Milford, America). An ACQUITY UPLC BEH-C18 (2.1 mm × 100 mm, 1.7 μm) column was applied for the separation work using 0.05% formic acid in water (A) and acetonitrile (B) as the mobile phase with a flow rate of 0.2 mL/min and a column temperature of 30 °C. The gradient elution program was as follows: 0-6 min, A 50%. The selected multiple reaction monitoring (MRM) modes in the negative ESI mode of the mass spectrometer were used for all the analytes, and the capillary voltage was set at 2.66 kV with a source temperature of 150 °C using nitrogen as the desolvation gas and cone gas. The CHL was used as an internal standard (IS). The MS parameters of FMN, BCA, and CHL are presented in Table S1.

#### 2.9.2. Preparation of Calibration Standards and Quality Control (QC) Samples

The same amounts of FMN and BCA were weighed and dissolved in acetonitrile to make 10 μg/mL of the stock solution. Then it was diluted with acetonitrile to a series of working solutions in the range of 2.5 to 2500 ng/mL. QC working solutions were set at concentrations of 10, 500, and 1500 ng/mL. The accurately weighed CHL was dissolved in acetonitrile and diluted to the scale of the IS solution (1054 ng/mL).

Then, 40 μL of the working solutions, 40 μL of the IS solutions, and 320 μL of acetonitrile were added to 100 μL of blank rat plasma to make the calibration standards with nominal concentrations in the range of 0.25-250.00 ng/mL for FMN and BCA. The QC samples with concentrations of 1.00, 50.00, and 150.00 ng/mL were prepared by the same procedure as above.

#### 2.9.3. Preparation of Plasma Sample

All plasma samples were stored at −20 °C and thawed at room temperature. To precipitate the protein, a 100 μL plasma sample was precisely aspirated, adding 40 μL of the IS solutions and 260 μL of acetonitrile, and vortexing for 3 min. Then the mixture was centrifuged at 13,000 rpm for 10 min and the 5 μL supernatant was injected for = UPLC-MS/MS analysis.

#### 2.9.4. Method Validation

The established UPLC-MS/MS analytical method was validated according to the bioanalytical method guidelines suggested by Chinese Pharmacopoeia 2020 [16]. The specificity was evaluated by comparing the chromatograms of the blank plasma sample, the blank plasma sample spiked with the analytes and IS, and the plasma sample after oral administration.

The standard calibration curves were plotted by the peak area ratio of individual analytes to IS (Y) versus the nominal analyte concentrations (X) and fitted by a weighted (1/X^2^) least-square regression with the required correlation coefficient (r) higher than 0.99. The lowest concentration of the curve with a signal-to-noise ratio of 10 was the lower limit of quantification (LLOQ).

Three levels (1, 50, and 150 ng/mL) of QC samples were analyzed in 6 replicates on one day for intra-day accuracy and precision and on three separate days for inter-day accuracy and precision. The precision and accuracy were expressed as relative standard deviation (RSD), not exceeding 15% and within ±15%, respectively.

The extraction recoveries were calculated according to the peak area ratios of the analytes in the pre-extraction and post-extraction spiked samples at three QC levels (1.00, 50.00, and 150.00 ng/mL). The peak areas of the analytes in the post-treated blank plasma and standard solution were compared to evaluate the matrix effects. Each experiment was measured 6 times in parallel.

Three level QC samples (1, 50, and 150 ng/mL) were analyzed to assess the stability of the analyte. The short-term stability was assessed by analyzing QC samples stored at room temperature for 24 h were analyzed for the short-term stability and stored at −20 °C for 20 days for long-term stability. Samples subjected to three freeze-and-thaw cycles were measured for freeze–thaw stability (−20 °C).

### 2.10. Tissue Distribution of β-CD/CSE Inclusion Complex

The contents of FMN and BCA in the tissues of rats were also determined by the above-established UPLC-MS/MS method.

#### 2.10.1. Preparation of Calibration Standard Samples

Using the above analyte stock solutions and the IS solution, the working solutions were prepared at different concentrations for the different analytes: 10.00–1000.00 ng/mL in the heart and brain; 10.00–800.00 ng/mL in the spleen; 10.00–2500.00 ng/mL in the lungs; 100.00–5000.00 ng/mL in the kidneys; and 100.00–7500.00 ng/mL in the liver.

The calibration standards were obtained by adding 120 μL working solutions and 60 μL IS solutions to 300 μL of processed blank tissue homogenate. The standard concentration ranges of FMN and BCA in different blank tissue homogenates were as follows: 1.00–100.00 ng/mL in the heart and brain; 1.00–80.00 ng/mL in the spleen; 1.00–250.00 ng/mL in the lungs; 10.00–500.00 ng/mL in the kidneys; and 10.00–750.00 ng/mL in the liver.

#### 2.10.2. Preparation of Tissue Sample

The tissue was washed with normal saline to remove the residual blood, the water on the surface of the tissue was dried, and the tissue was weighed, cut into small pieces, and put into a centrifuge tube. Then, it was added to normal saline of the same amount as the tissue and ground into a homogenate. Then, 300 μL of the tissue homogenate was placed into a centrifuge tube, adding 60 μL of IS and 840 μL of acetonitrile to precipitate the protein. After centrifuging at 13,000 rpm for 10 min, the supernatant was collected and centrifuged again. Finally, the obtained supernatant was taken for subsequent analysis.

## 3. Results and Discussion

### 3.1. The Isoflavones Content Changes in Sprouted Chickpeas

**Figure 1:**
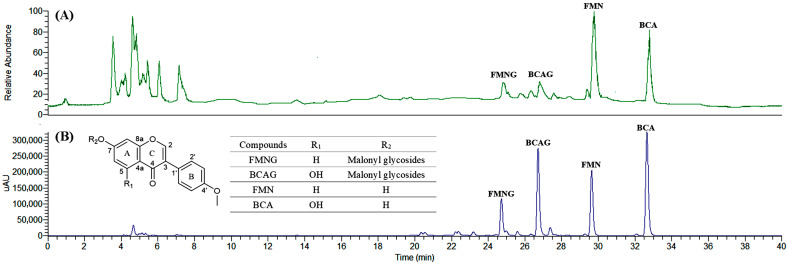
The base peak chromatography (BPC) in the positive ion mode (**A**) and HPLC chromatogram (**B**). (HPLC conditions: 5 μm Welch Ultimate XB-C18, 250 mm × 4.6 mm i.d; 0.05% formic acid in water and acetonitrile as the mobile phase; a column temperature of 30 °C; and a flow rate of 1.0 mL/min).

The isoflavones in sprouted chickpeas were first analyzed by UHPLC-ESI-IT-MS in positive ion mode. The base peak chromatography (BPC) and HPLC chromatogram of the sprouted chickpeas are shown in Figure 1A,B. Two isoflavones and their glycosides were isolated and identified in sprouted chickpeas. They are formononetin malonyl glycoside (FMNG) with [M+H]^+^ 517.02, biochanin A malonyl glycoside (BCAG) with [M+H]^+^ 533.06, FMN with [M+H]^+^ 269.24, and BCA with [M+H]^+^ 285.21 [17]. The MS spectra and molecular structure of these four compounds are shown in Table S2 and Figure S1. FMN and BCA are the main isoflavone aglycones in the chickpea with substitution difference at the C5 position of the A ring. According to Figure 1A, the FMNG and BCAG showed a low ionization efficiency as compared to FMN and BCA. A similar phenomenon was also found by Gao et al. [18] This may be attributed to the fact that flavonoid glycosides are more likely to break the glycosidic bonds and form aglycone fragments after ionization by mass spectrometry [18]. Many studies have demonstrated that isoflavone aglycones have stronger bioavailability than their glycoside derivatives [19,20]. Hence, FMN and BCA were selected to evaluate changes in isoflavone content during the chickpea sprouting and bioavailability test.

**Figure 2:**
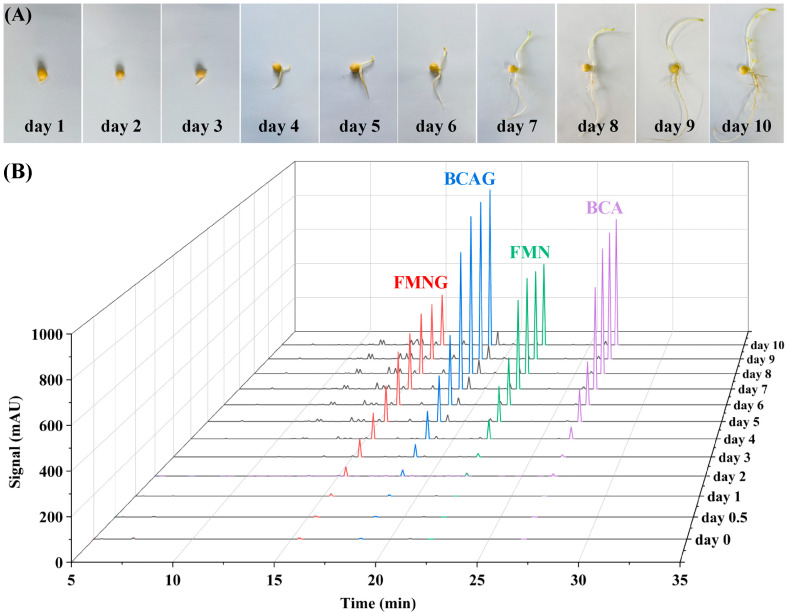
The (**A**) appearance of chickpea and (**B**) HPLC profiles of chickpea extracts with different germination stages.

The appearance of chickpea and HPLC profiles of chickpea extracts with different germination stages are shown in Figure 2. In the process of chickpea germination and growth, the germ and radicle grew continuously, and the cotyledons shrank gradually, accompanied by the change in components [21,22]. The content of four isoflavones all increased with germination. The peak area values of FMNG, BCAG, FMN, and BCA with different germination days are shown in Table S3. The content of glycoside derivatives of isoflavones (FMNG and BCAG) increased faster than the corresponding aglycones (FMN and BCA) in the initial stage (0 to 3 days). However, from day 4 to 8, the content of FMN and BCA increased rapidly, and the chickpea germs also grew rapidly. After the 8th day, the content became stable for BCAG and BCA and slightly decreased for FMNG and FMN. The trend of content variation of the two flavonoid glycosides appeared to be the same as their corresponding aglycones. Gao et al. found that the content of FMN and BCA in chickpeas reached the maximum on the 10th day under completely dark culture conditions; under the condition of continuous light, the best germination time of chickpea was 8 days, and the content of isoflavones was higher than that of chickpea incubated in darkness [18]. Wu et al. found that FMN and BCA in chickpeas no longer increased on the 4th day of germination [7]. These differences may be related to different cultivation conditions or origins of chickpeas. Nevertheless, it can be concluded that the increase in isoflavones is due to the growth of chickpea germ and radicle. We determined the FMN and BCA contents of each part of the sprouted chickpea on day 8 (Figure S2). As expected, most of the isoflavones were concentrated in the germ and radicle. Overall, the 8th day of sprouted chickpeas was selected for the preparation of CSE.

Germinated chickpeas (the 8th day) were defatted with petroleum ether and extracted with aqueous ethanol to obtain an isoflavone-rich CSE. The total flavonoid content measured by the AlCl_3_ chromogenic method in CSE was 73.8 mg/g (7.38% in mass). On the other hand, FMN and BCA determined by the above HPLC method were 22.1 mg/g and 34.9 mg/g, respectively. The amount of total flavonoids measured was larger than the sum of FMN and BCA measured using the HPLC method. This may be due to the different methods used and the presence of other flavonoids in the extracts.

### 3.2. Optimizing the Preparation of CSE/β-CD Inclusion Complexes

The poor water solubility of isoflavones will reduce their bioavailability and pharmacological activities [13,23]. Therefore, improving the water solubility of isoflavones is a feasible solution to enhance their biological activity. Research showed that by forming an inclusion complex, β-CD could enhance the isoflavones’ water solubility [24]. Based on a previous study, attempts were made to increase the water solubility of CSE isoflavones by forming inclusion complexes with β-CD. There are many methods, such as the saturated aqueous solution method and grinding method, that have been successfully used to fabricate cyclodextrin inclusion complexes [9,15]. In this study, a saturated aqueous solution method was used to prepare the CSE/β-CD inclusion complexes, which have the advantages of simplicity and high efficiency [25]. The preparation conditions of the inclusion complexes were optimized using the inclusion ratio of FMN and BCA as the optimization parameters (Figure S3).

The factors including the mass ratio, temperature, reaction time, and mixing speed were evaluated (Figure S4). When the mass ratio of β-CD/CSE was 4:1, the temperature was 40 °C, and stirring occurred at 400 rpm for 2 h, the inclusion ratio of both FMN and BCA was higher. Among these factors, the inclusion temperature (A), inclusion time (B), and mixing speed (C) were selected, and a three-factor and three-level orthogonal test (Table S4) was designed according to the L9(34) table. The orthogonal experiment results of the inclusion ratio of FMN and BCA are shown in Tables S5 and S6. The influences of three factors on the inclusion ratio of the two isoflavones were in the same order: A > B > C. However, the FMN and BCA in CSE exhibited different inclusion properties with β-CD, and BCA showed a much higher inclusion ratio than FMN. According to previous studies, the B ring of flavonoids (FMN/BCA) could be inserted into the hydrophobic cavity of β-CD in a stoichiometric ratio of 1:1 [11,12,13]. The extra OH group at the C5 position of BCA may promote hydrogen bond formation, thereby increasing the inclusion ratio. The orthogonal test verified the optimized conditions obtained from the single-factor experiment. Under the optimal conditions, the inclusion ratios of FMN and BCA were 53.31% and 81.79%, respectively. The drug loading of FMN and BCA in the CSE/β-CD inclusion complex was 5.2 mg/g and 17.9 mg/g, respectively.

### 3.3. Characterization of the CSE/β-CD Inclusion Complexes

The UV spectra of β-CD, CSE, the CSE/β-CD inclusion complex, and the physical mixture of CSE/β-CD in the wavelength range of 200–400 nm are shown in Figure S4. β-CD has no absorption in this range, and all other CSE-containing mixtures show the characteristic absorption peak of isoflavones at approximately 260 nm. Compared with the physical mixture, the maximum absorption peaks of isoflavones showed a slight blue shift in the inclusion complex. This suggests that the microenvironment of the chromophore in isoflavones is changed by the interactions with β-CD, including hydrogen bonding and/or hydrophobic interactions, rather than simple physical mixing [13,25].

The FT-IR spectra of β-CD, CSE, the CSE/β-CD inclusion complex, and the physical mixture of CSE/β-CD in the range of 4000-400 cm^−1^ are collected (Figure S5). Prominent absorption bands in the FT-IR spectrum of β-CD are observed at 3405, 2925, 1637, 1158, 1079, and 1028 cm^−1^. CSE showed its primary absorption bands at 3383, 2930, 1679, and 1516 cm^−1^. The physical mixture of CSE/β-CD contains all the above characteristic absorption bands, indicating that CSE and β-CD both retain their basic chemical structures. The CSE/β-CD inclusion complex had a similar FT-IR spectrum to β-CD with the characteristic carbonyl group absorption peak of the CSE at 1679 cm^−1^ disappearing, and the benzene ring skeleton vibration at 1516 cm^−1^ weakened. It is speculated that the benzene ring of isoflavones enters the hydrophobic cavity of β-CD to form the inclusion complex, thereby shielding the signal of the carbonyl group on the C ring. Overall, no new functional groups with covalent bonds were found, indicating that the formation of the inclusion complex is based on weak intermolecular interactions [26].

**Figure 3:**
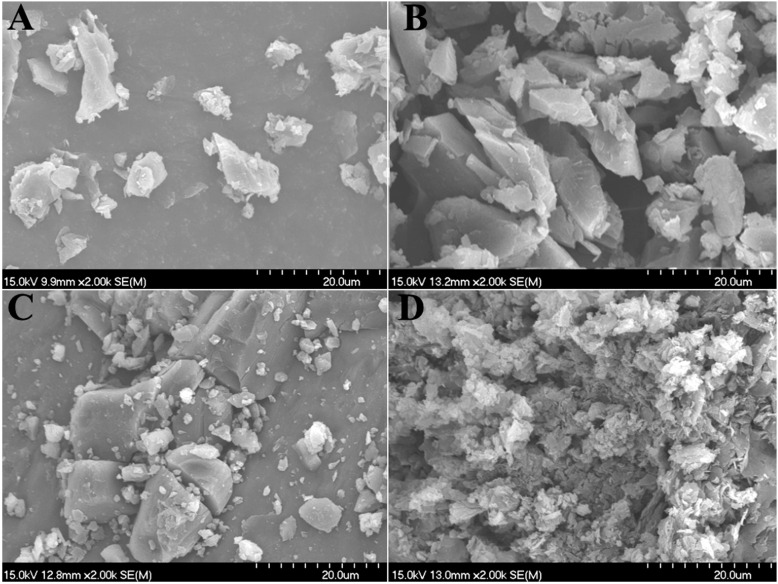
SEM micrographs of chickpea sprout extracts (CSE) (**A**), β-CD (**B**), physical mixture (**C**), and inclusion complex (**D**).

The SEM analysis reflects the changes in the surface morphology of the materials (Figure 3). The SEM picture of CSE with a magnification of 2000 showed irregular granules with small volumes, and β-CD showed irregular blocks with large volumes. In the physical mixture, the particle structures of these two different forms could be observed with obvious separation. However, the CSE/β-CD inclusion complex showed a dense and uniform lamellar structure. It is speculated that the rough outer layer and dense structure of the CSE/β-CD inclusion complex are due to the presence of amorphous cyclodextrins, which form complexes with isoflavonoids in CSE through hydrophobic inclusion and hydrogen bonding interactions [27,28].

### 3.4. In Vitro Dissolution Profiles in Artificial Digestion Juice

**Figure 4:**
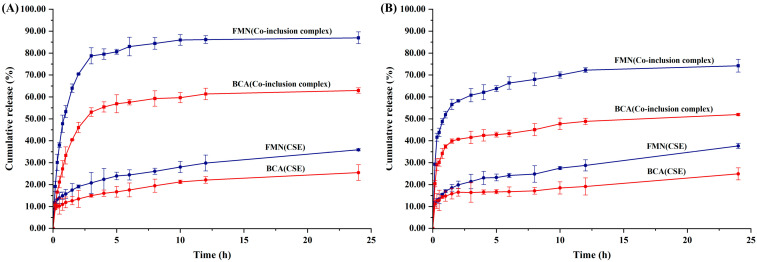
The cumulative dissolution rate of FMN and BCA in artificial gastric juice (**A**) and artificial intestinal juice (**B**).

Artificial gastric juice and artificial intestinal juice were applied as release media to determine the dissolution properties of FMN and BCA in the CSE and CSE/β-CD inclusion complex (Figure 4). For the same compound, the release rates in artificial gastric juice were better than those in artificial intestinal juice due to the differences in physical properties of the release media and the surrounding microenvironment [29]. Interestingly, the cumulative release of FMN was higher than that of BCA in both of the media. The release rate of FMN and BCA in the inclusion complex was faster in the release medium with a higher cumulative release rate than CSE. It indicates that by forming the CSE/β-CD inclusion complex, the solubility and dissolution properties of FMN and BCA were significantly improved. Different mathematical models are applied to correlate the cumulative release curves [30]. The results presented in Table S7 show that the Higuchi model is best suited for isoflavones release profiles from CSE, while the Weibull model is best suited for these from the inclusion complexes. This suggests that the release of isoflavones from the CSE and the inclusion complexes follows different mass transfer mechanisms [31]. Overall, the CSE/β-CD inclusion complex exhibited good dissolution properties for isoflavones in artificial digestion juice, suggesting that the isoflavones therein may also have good bioaccessibility and absorption properties in vivo.

### 3.5. In Vivo Oral Pharmacokinetics in the Rat

#### 3.5.1. Validation of UPLC-MS/MS Method for Quantification of FMN and BCA

**Figure 5:**
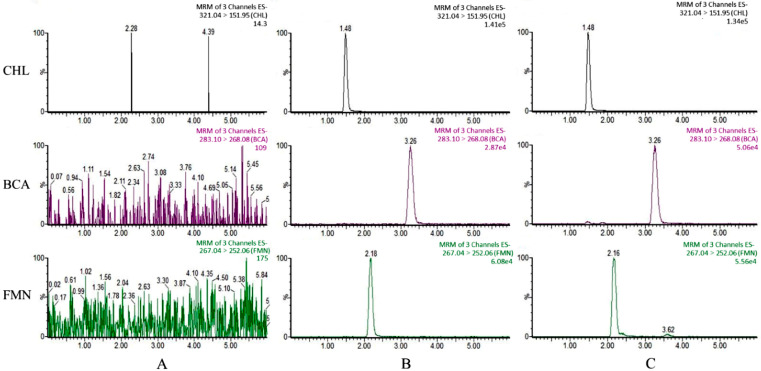
Typical MRM chromatograms of (**A**) blank plasma, (**B**) blank plasma spiked with FMN, BCA and CHL (the line was shown in purple), and (**C**) representative plasma sample at 15 min after oral administration of the CSE spiked with the IS (the line was shown in green).

The FMN and BCA distributed in plasma and tissues were quantified by a UPLC-MS/MS method. Figure 5 shows the chromatograms of blank plasma, blank plasma spiked with analytes and IS, and a plasma sample at 15 min spiked with IS after oral administration of the CSE. The analytes and IS were well separated with no interference by endogenous substances in the plasma. The linear ranges, regression equation, correlation coefficient (r) (>0.99), and LLOQs of the two analytes in plasma are shown in Table S8. All the values including the intra- and inter-day precisions (RSD) of the analytes, the accuracy (RE), the extraction recoveries, and the matrix effect were within the acceptable range. After 24 h at 25 °C, 30 days at −20 °C, and three freeze–thaw cycles, the corresponding precision and accuracy for stability were in the range of 2.26–13.57% and −3.79–8.47%, demonstrating the stability of all analytes in plasma under the abovementioned conditions.

#### 3.5.2. Pharmacokinetic Profiles in Rat Plasma

**Figure 6:**
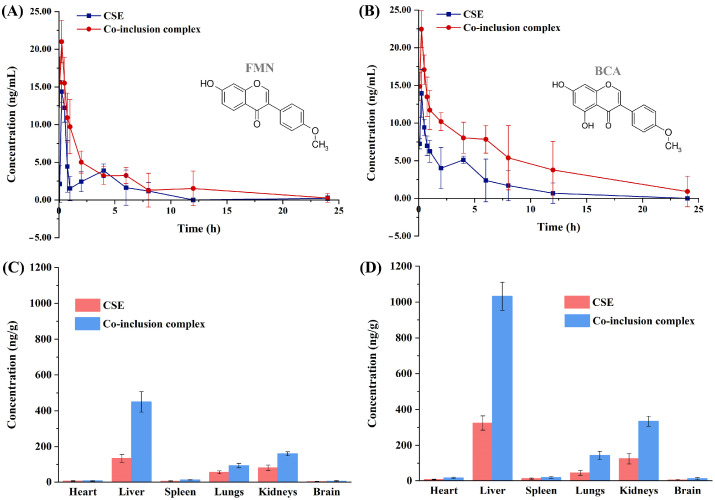
Mean plasma concentration-time profiles of FMN (**A**) and BCA (**B**) in rats after oral administration of CSE and inclusion complex; distribution in the tissue of FMN (**C**) and BCA (**D**) in rats after oral administration of CSE and inclusion complex.

Figure 6A,B exhibit the mean plasma concentration–time profiles of the two isoflavones FMN and BCA. The concentrations of FMN and BCA in rat plasma reached the maximum at 15 min after being administered orally. The maximum plasma concentration (C_max_) of the two isoflavones in the plasma of rats fed the inclusion complex was significantly higher than that of rats fed CSE. This may be because β-CD contains multiple hydrophilic hydroxyl groups, which causes the inclusion complex to have better wettability and solubility, and drug molecules pass through biological cell membranes more easily [32,33].

PKSolver software was applied to calculate the main pharmacokinetic parameters with the results shown in Table 1. Whether the rats were given the CSE or the inclusion complex, the concentrations of FMN and BCA in rat plasma all reached the peak after 15 min, indicating that the absorption rate of FMN and BCA in rats was very fast. Compared with the rats fed the CSE, the rats fed the inclusion complex had higher C_max_, longer elimination half-lives (t_1/2_), larger areas under the concentration–time curve (AUC), and lower apparent volumes of distribution (Vd) and plasma clearance (CL). This result showed that by forming an inclusion complex, the absorption degree of FMN and BCA in rats increased, and the elimination rate was reduced. This caused the content of FMN and BCA in rats to be higher and have a longer retention time. The relative bioavailability of the two isoflavones in the inclusion complex relative to the CSE was calculated using the ratio of the AUC. The relative bioavailability of FMN and BCA was 171.6% and 270.7%, respectively. The oral bioavailability of FMN and BCA in the CSE/β-CD inclusion complex was significantly improved. It is reported that β-CD and its derivatives can inhibit the activity of P-glycoprotein, which is closely related to the bioavailability of oral drugs. The decrease in P-glycoprotein activity can reduce the excretion of drugs from cells [34].

### 3.6. Tissue Distribution In Vivo

The linear ranges, regression equation, correlation coefficient r (>0.99), and LLOQs of FMN and BCA in tissues are shown in Table S9. The distribution of FMN and BCA in various tissues of rats is shown in Figure 6C,D. The contents of FMN and BCA in rat tissues were much higher than those in plasma, and two peaks were observed in the concentration–time curve, which may be caused by the distribution re-absorption or entero-hepatic recirculation of drugs in rats [35]. The two isoflavones had been detected in various tissues, which proved that they were widely distributed. From Figure 6, it can be seen intuitively that there is no obvious change in the tissue distribution trend of FMN and BCA in either the CSE or the inclusion complex. The distribution characteristics of the contents of these two isoflavones in rat tissues were liver > kidneys > lungs > spleen > heart > brain, and the content in the liver and kidney was significantly higher than that in other tissues, which may be because the liver and kidney are the main metabolic and excretory organs [36]. The content of FMN and BCA in the inclusion complex in the tissues was significantly higher than that of the CSE. It was speculated that after the inclusion complex made of CSE, the absorption rate of these two isoflavones in the tissue was accelerated, and the metabolism rate was slowed down, leading to a significant increase in the accumulation in the tissues.

## 4. Conclusions

The isoflavones content in chickpeas increased through sprouting, and the content distribution of isoflavones in different parts of sprouted chickpeas was comparatively determined. The CSE/β-CD inclusion complex was prepared, which significantly improved the sustained release in the simulated digestive model. The UPLC-MS/MS analysis method for the simultaneous determination of FMN and BCA in plasma or tissues was also established. The experimental results showed that the concentrations of FMN and BCA in the inclusion complex could reach higher levels in the plasma or tissues of rats, indicating that β-CD can effectively improve the poor solubility of isoflavones and improve their bioavailability in rats. The study provides a feasible strategy for the solubilization of isoflavones from chickpeas and improving their bioavailability.

## Figures and Tables

**Table 1:** Pharmacokinetic parameters of formononetin (FMN) and biochanin A (BCA) in rats after oral administration of chickpea sprout extract (CSE) and inclusion complex.

Parameter	FMN (CSE)	FMN (Inclusion Complex)	BCA (CSE)	BCA (Inclusion Complex)
Ke/h^−1^	0.23 ± 0.12	0.20 ± 0.13	0.15 ± 0.02	0.11 ± 0.04
t_1/2_/h	3.84 ± 2.10	4.51 ± 2.07	4.63 ± 0.88	7.33 ± 3.07
t_max_/h	0.25 ± 0.00	0.25 ± 0.00	0.25 ± 0.00	0.25 ± 0.00
C_max_(ng/mL)	14.37 ± 1.46	21.00 ± 2.81	13.94 ±3.17	22.45 ± 2.45
AUC_0→t_/(ng·h/mL)	29.99 ± 15.21	51.46 ± 12.29	37.59 ± 17.70	101.75 ± 45.29
AUC_0→∞_/(ng·h/mL)	36.96 ± 12.86	72.05 ± 25.70	61.89 ± 15.53	173.40 ± 56.27
Vd/L	722.51 ± 197.97	441.60 ± 140.88	1635.82 ± 92.84	896.37 ± 113.90
CL/(L/h)	148.14 ± 47.18	75.79 ± 23.47	252.24 ± 52.73	93.83 ± 29.01

## Data Availability

The data presented in this study are available within the manuscript.

## Supplementary Materials

The following supporting information can be downloaded at: https://www.mdpi.com/article/10.3390/pharmaceutics15122684/s1, Figure S1: The MS spectra of principal isoflavones (figures A, B, C, and D are the MS spectra of FMNG, BCAG, FMN, and BCA, respectively); Figure S2: The FMN and BCA contents of each part of germinated chickpea on day 8; Figure S3: Effect of (A) mass ratio, (B) temperature, (C) time, and (D) mixing speed on the inclusion ratio of formononetin (FMN) and biochanin A (BCA); Figure S4: UV spectra of CSE, β-CD, physical mixture, and inclusion complex; Figure S5: FT-IR spectra of (A) CSE, (B) β-CD, (C) physical mixture, and (D) inclusion complex; Table S1: The MS/MS parameter for FMN, BCA, and CHL; Table S2: Principal isoflavones in germinated chickpea; Table S3: Peak area of FMNG, BCAG, FMN, and BCA in chickpeas with different germination days; Table S4: Factors and levels of orthogonal experiments; Table S5: The orthogonal experiments result for FMN; Table S6: The orthogonal experiments result for BCA; Table S7: Results of curve fitting of in vitro release from the CSE and inclusion complex; Table S8: The regression equation, precision, accuracy, extraction recoveries, matrix effects, and stability of FMN and BCA in rat plasma; Table S9: The linear range, regress equations, and LLOQs of FMN and BCA.
