# Supplementary material for: Improving Bioaccessibility and Bioavailability of Isoflavone Aglycones from Chickpeas by Germination and Forming β-Cyclodextrin Inclusion Complexes
Source: Pharmaceutics. 2023 Nov 27;15(12):2684. doi: 10.3390/pharmaceutics15122684 (PMC10747479; doi:10.3390/pharmaceutics15122684)
Supplement: Supplementary file 1 [file pharmaceutics-15-02684-s001.zip › pharmaceutics-2674348-supplementary.pdf]

Improving bioaccessibility and bioavailability of isoflavone aglycones from chickpeas by germination and forming β-cyclodextrin inclusion complexes

Electronic Supplementary Information (ESI) available: [Table S1, S2, S3, S4, S5, S6, S7, S8, S9 and Figure S1, S2, S3, S4, S5].

Table S1. The MS/MS parameter for FMN, BCA and CHL.

Compounds	Molecular formula	Precursor ion (*m/z*)	Product ion (*m/z*)	Cone voltage (V)	Collision energy (V)
FMN	$C_{16}H_{12}O_4$	267.04	252.06	60	18
BCA	$C_{16}H_{12}O_5$	283.10	268.08	40	20
CHL (IS)	$C_{11}H_{12}Cl_2N_2O_5$	321.04	151.95	60	18

Table S2. Principal isoflavones in germinated chickpea.

Retention time (min)	$[M+H]^+$ (*m/z*)	Compounds	Molecular formula
24.72	517.02	Formononetin malonyl glycoside	$C_{25}H_{24}O_{12}$
26.72	533.06	Biochanin A malonyl glycoside	$C_{25}H_{24}O_{13}$
29.64	269.24	Formononetin	$C_{16}H_{12}O_4$
32.65	285.21	Biochanin A	$C_{16}H_{12}O_5$

Table S3. Peak area of FMNG, BCAG, FMN, and BCA in chickpeas with different germination days.

Time (h)	Peak area			
	FMNG	BCAG	FMN	BCA
0	38.50	35.00	0.00	0.00
0.5	42.00	34.20	0.00	17.00
1	92.40	49.80	13.40	14.30
2	391.60	275.20	144.40	104.20
3	790.30	544.80	188.40	128.60
4	1184.20	1260.70	993.00	639.40
5	1731.00	2108.00	1772.80	1753.10
6	2559.40	3317.60	2526.90	2405.20
7	2735.30	5994.90	4865.90	5790.40
8	3018.60	7919.30	5336.40	6933.20
9	2684.40	8040.40	5096.10	7836.90
10	2551.20	7960.50	4905.80	8149.30

Table S4. Factors and levels of orthogonal experiments.

Level	Factor		
	A, Temperature (°C)	B, Time (h)	C, Mixing speed (rpm)
1	30	0.5	400
2	40	1	600
3	50	2	800

Table S5. The orthogonal experiments result for FMN.

Number	A, Temperature (°C)	B, Time (h)	C, Mixing speed (rpm)	Blank	Inclusion rate (%)
1	1 (30)	1 (0.5)	1 (400)	1	36.94 ± 0.90
2	1	2 (1)	2 (600)	2	40.92 ± 0.97
3	1	3 (2)	3 (800)	3	39.40 ± 0.30
4	2 (40)	1	2	3	48.27 ± 0.71
5	2	2	3	1	51.99 ± 0.99
6	2	3	1	2	53.31 ± 1.67
7	3 (50)	1	3	2	33.53 ± 1.62
8	3	2	1	3	38.53 ± 1.43
9	3	3	2	1	39.26 ± 0.48
K1	117.26	118.74	128.78	128.20	
K2	153.57	131.44	128.44	127.76	
K3	111.31	131.96	124.92	126.19	
k1	39.09	39.58	42.93	42.73	
k2	51.19	43.81	42.81	42.59	
k3	37.10	43.99	41.64	42.06	
R	14.09	4.41	1.29	0.67	

Table S6. The orthogonal experiments result for BCA.

Number	A, Temperature (°C)	B, Time (h)	C, Mixing speed (rpm)	Blank	Inclusion rate (%)
1	1 (30)	1 (0.5)	1 (400)	1	68.11 ± 0.28
2	1	2 (1)	2 (600)	2	71.35 ± 0.76
3	1	3 (2)	3 (800)	3	71.56 ± 0.44
4	2 (40)	1	2	3	75.94 ± 0.74
5	2	2	3	1	80.50 ± 1.00
6	2	3	1	2	81.79 ± 0.76
7	3 (50)	1	3	2	65.74 ± 0.34
8	3	2	1	3	69.63 ± 0.02
9	3	3	2	1	68.99 ± 0.22
K1	211.01	209.79	219.53	217.60	
K2	238.23	221.48	216.27	218.88	
K3	204.36	222.34	217.80	217.12	
k1	70.34	69.93	73.18	72.53	
k2	79.41	73.83	72.09	72.96	
k3	68.12	74.11	72.60	72.37	
R	11.29	4.18	1.09	0.59	

Table S7. Results of curve fitting of in vitro releasing from the CSE and inclusion complex.

Models	Regression co-efficient							
	Artificial gastric juice				artificial intestinal juice			
	CSE		inclusion complex		CSE		inclusion complex	
	FMN	BCA	FMN	BCA	FMN	BCA	FMN	BCA
Zero Order	0.851	0.845	0.376	0.432	0.864	0.862	0.520	0.543
First Order	0.885	0.870	0.846	0.611	0.889	0.865	0.675	0.594
Higuchi	<u>0.992</u>	<u>0.984</u>	0.653	0.710	<u>0.982</u>	<u>0.902</u>	0.772	0.768
Ritger-Peppas	0.988	0.984	0.819	0.841	0.975	0.862	0.929	0.904
Hixon-Crowell	0.874	0.862	0.744	0.547	0.881	0.864	0.625	0.577
Weibull	0.984	0.974	<u>0.912</u>	<u>0.903</u>	0.975	0.894	<u>0.950</u>	<u>0.912</u>

Table S8. The regression equation, precision, accuracy, extraction recoveries, matrix effects and stability of FMN and BCA in rat plasma.

**Compounds**		**FMN**			**BCA**		
Calibration range (ng/mL)		0.25-250.00			0.25-250.00		
Regression equation		Y=0.0468X + 0.0235			Y=0.0450X - 0.0226		
Correlation coefficient		0.9995			0.9993		
LLOQs (ng/mL)		0.25			0.25		
Spiked conc. (ng/mL)		1	50	150	1	50	150
Measured conc. (ng/mL)		1.04 ± 0.03	47.43 ± 1.98	149.56 ± 6.12	1.04 ± 0.04	48.18 ± 2.62	141.84 ± 5.01
Intra-day	Precision (RSD, %)	3.12	4.18	4.09	4.13	5.45	3.53
	Accuracy (RE, %)	3.81	-5.15	-0.29	4.11	-3.64	-5.44
Inter-day	Precision (RSD, %)	5.09	7.32	3.60	5.53	6.41	4.65
	Accuracy (RE, %)	2.72	-1.09	0.39	5.13	2.53	-0.11
Recovery %		85.74 ± 5.77	86.12 ± 4.23	98.00 ± 7.58	86.76 ± 11.45	87.19 ± 10.71	83.13 ± 8.84
Matrix effect %		109.13 ± 4.26	119.07 ± 7.86	101.44 ± 9.93	88.63 ± 9.33	113.83 ± 12.92	116.18 ± 9.02
25 ℃ for 24 h	Precision (RSD, %)	3.54	9.12	5.89	5.04	8.33	7.19
	Accuracy (RE, %)	1.23	-1.90	-3.79	7.02	-0.66	1.35
-20 ℃ for 30 days	Precision (RSD, %)	4.04	6.82	2.90	5.52	5.49	4.47
	Accuracy (RE, %)	3.76	-2.69	-0.43	2.95	3.17	1.90
Three freeze-thaw	Precision (RSD, %)	7.42	13.57	2.26	3.25	5.63	5.26
	Accuracy (RE, %)	0.54	-0.43	2.20	8.47	-0.26	-2.19

Table S9. The linear range, regress equations, and LLOQs of FMN and BCA.

Compounds	Tissue	Calibration range (ng/mL)	Regression equation	Correlation coefficient (r)	LLOQs (ng/mL)
FMN	Heart	1-100	Y=0.1695X + 0.1636	0.9991	1
	Liver	10-750	Y=0.0650X + 0.9520	0.9996	10
	Spleen	1-80	Y=0.1393X + 0.2404	0.9992	1
	Lungs	1-250	Y=0.0871X + 0.2884	0.9998	1
	Kidneys	10-500	Y=0.0559X + 0.3989	0.9995	10
	Brain	1-100	Y=0.0881X + 0.1637	0.9992	1
BCA	Heart	1-100	Y=0.1269X + 0.1711	0.9989	1
	Liver	10-750	Y=0.0521X + 0.9090	0.9999	10
	Spleen	1-80	Y=0.1046X + 0.2248	0.9992	1
	Lungs	1-250	Y=0.0618X + 0.7329	0.9995	1
	Kidneys	10-500	Y=0.0468X + 0.5373	0.9999	10
	Brain	1-100	Y=0.0705X + 0.0350	0.9993	1

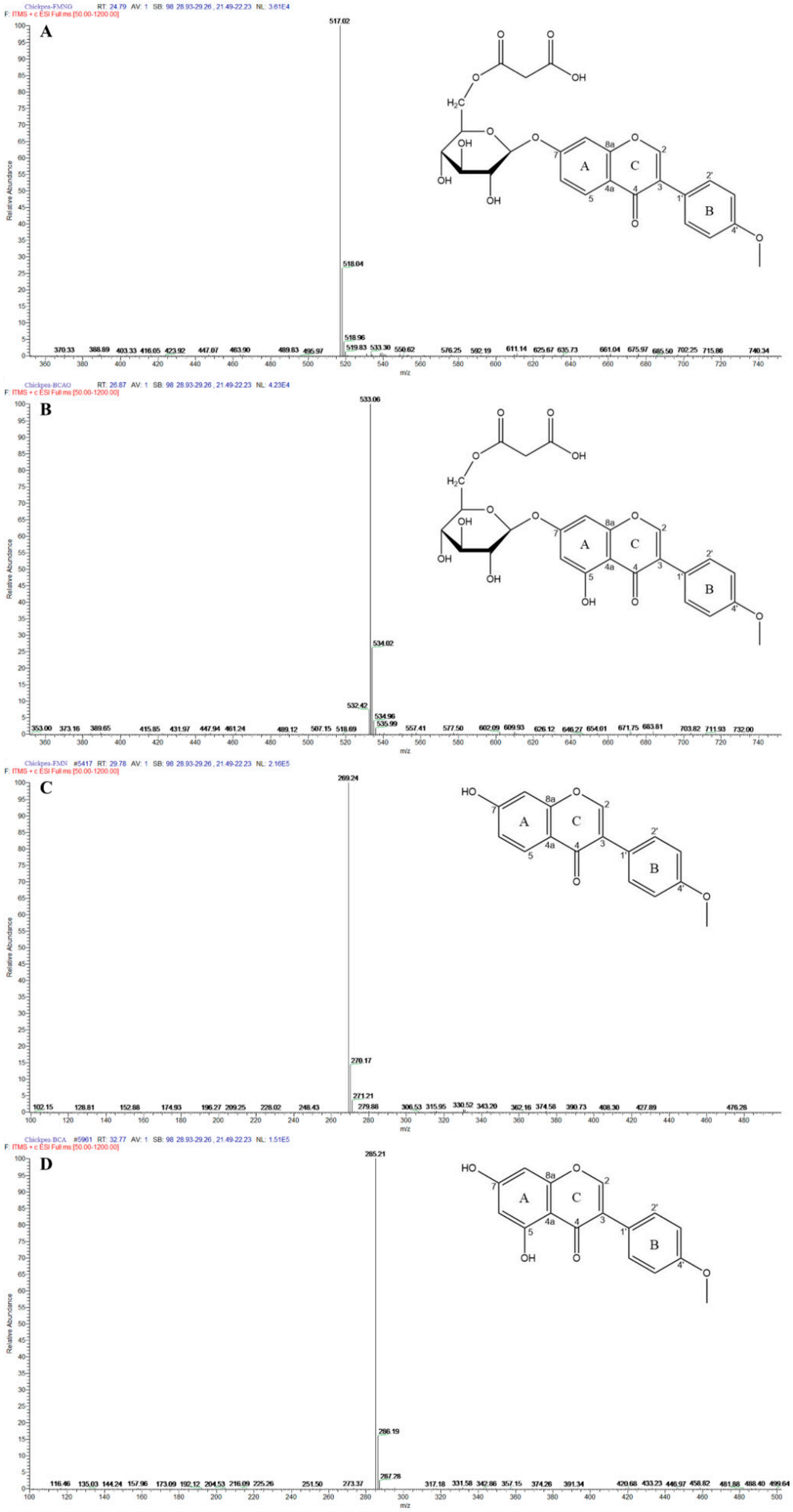

Figure S1. The MS spectra of principal isoflavones (figures A, B, C and D are the MS spectra of FMNG, BCAG, FMN and BCA, respectively).

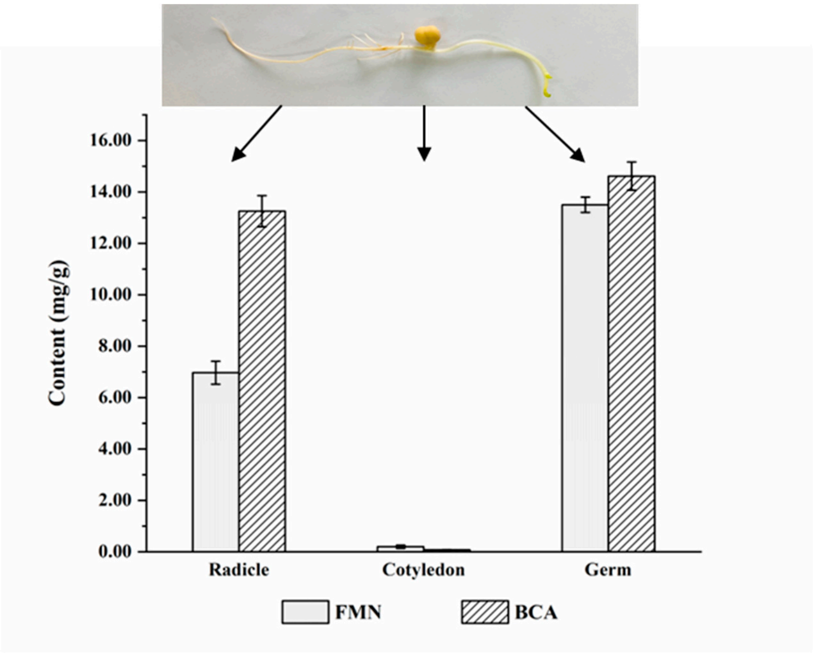

Figure S2. The FMN and BCA contents of each part of germinated chickpea on day 8.

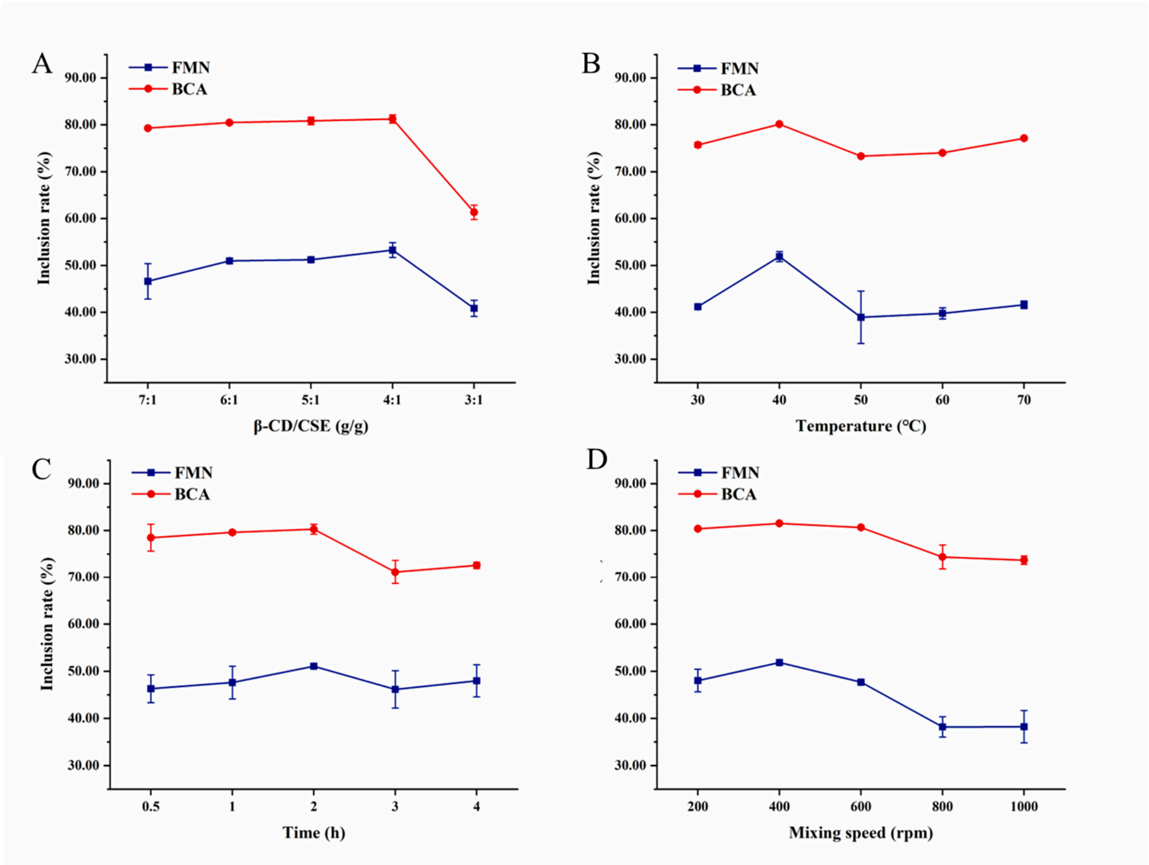

Figure S3. Effect of (A) mass ratio, (B) temperature, (C) time and (D) mixing speed on the inclusion rate of formononetin (FMN) and biochanin A (BCA).

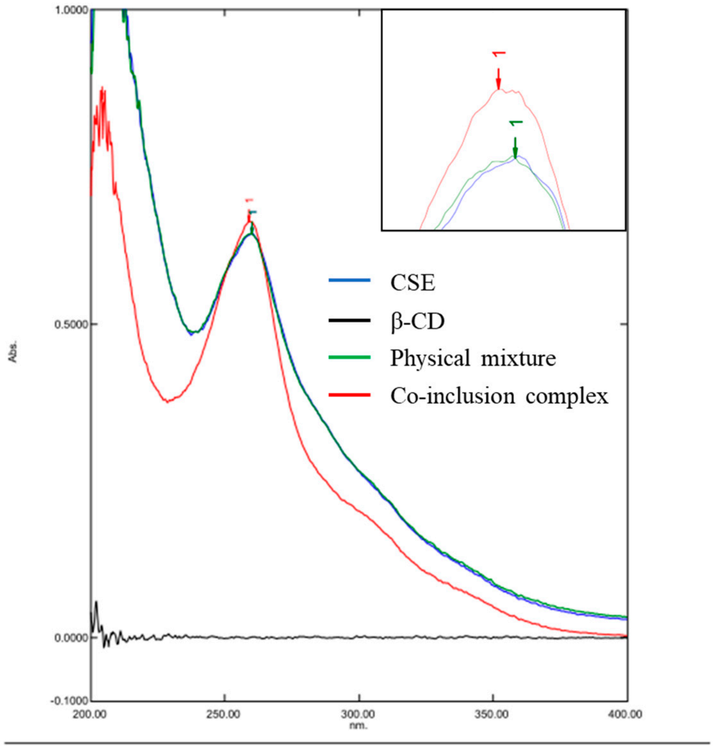

Figure S4. UV spectra of CSE, β-CD, physical mixture and inclusion complex.

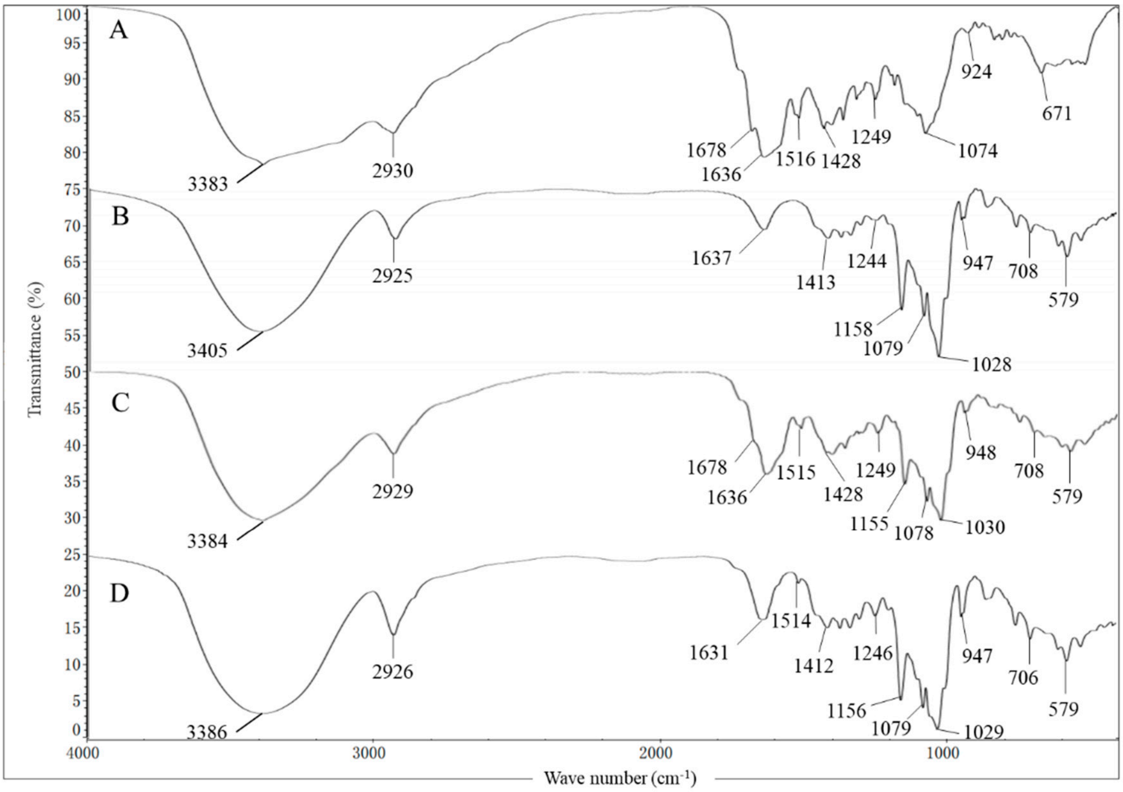

Figure S5. FT-IR spectra of (A) CSE, (B) β-CD, (C) physical mixture and (D) inclusion complex.
